# Respiratory Event-Induced Blood Pressure Oscillations Vary by Sleep Stage in Sleep Apnea Patients

**DOI:** 10.1155/2023/8787132

**Published:** 2023-06-15

**Authors:** Yao Shun Chaung, Raichel M. Alex, Mahrshi Jani, Donald E. Watenpaugh, Radana Vilimkova Kahankova, Scott A. Sands, Khosrow Behbehani

**Affiliations:** ^1^School of Biomedical Informatics, University of Texas Health Science Center at Houston, Houston, Texas, USA; ^2^Division of Sleep and Circadian Disorders, Brigham and Women's Hospital, Boston, MA, USA; ^3^Bioengineering Department, University of Texas at Arlington, Arlington, Texas, USA; ^4^Department of Cybernetics and Biomedical Engineering, VSB-Technical University of Ostrava, Ostrava, Czech Republic

## Abstract

Obstructive sleep apnea (OSA) pathologically stresses the cardiovascular system. Apneic events cause significant oscillatory surges in nocturnal blood pressure (BP). Trajectories of these surges vary widely. This variability challenges the quantification, characterization, and mathematical modeling of BP surge dynamics. We present a method of aggregating trajectories of apnea-induced BP surges using a sample-by-sample averaging of continuously recorded BP. We applied the method to recordings of overnight BP (average total sleep time: 4.77 ± 1.64 h) for 10 OSA patients (mean AHI: 63.5 events/h; range: 18.3-105.4). We studied surges in blood pressure due to obstructive respiratory events separated from other such events by at least 30 s (274 total events). These events increased systolic (SBP) and diastolic (DBP) BP by 19 ± 7.1 mmHg (14.8%) and 11 ± 5.6 mmHg (15.5%), respectively, relative to mean values during wakefulness. Further, aggregated SBP and DBP peaks occurred on average 9 s and 9.5 s after apnea events, respectively. Interestingly, the amplitude of the SBP and DBP peaks varied across sleep stages, with mean peak ranging from 128.8 ± 12.4 to 166.1 ± 15.5 mmHg for SBP and from 63.1 ± 8.2 to 84.2 ± 9.4 mmHg for DBP. The aggregation method provides a high level of granularity in quantifying BP oscillations from OSA events and may be useful in modeling autonomic nervous system responses to OSA-induced stresses.

## 1. Introduction

Sleep apnea is a neurorespiratory disorder manifested by repetitive restriction or cessation of breathing during sleep [1, 2]. Sleep apnea is categorized as obstructive, central, and mixed apnea [3]. Obstructive sleep apnea (OSA) is far more prevalent than other types and is estimated to affect more than 9% of the U.S. adult population [4]. Currently, a simple count of apnea and hypopnea events per hour of sleep, called apnea-hypopnea index (AHI), is the standard clinical metric of sleep-disordered breathing severity (SDB). When 5 ≤ AHI < 15 events/h, the disease severity is classified as mild, 15 ≤ AHI < 30 events/h is moderate, and 30 ≤ AHI events/h represents severe sleep apnea.

Sleep apnea causes or contributes to multiple cardiovascular disorders and cognitive deficiencies [5, 6]. In particular, OSA has been associated with hypertension [7]. Multiple studies have shown that OSA elevates blood pressure (BP) during awake hours [8] and diminishes or eliminates the normal overnight dipping pattern in BP [9, 10]. While there is strong association between OSA and hypertension, the mechanisms of this relationship are not fully understood [10–12]. Noninvasive beat-to-beat BP measuring devices [13] allow the measurement of nocturnal BP oscillations. Such account of nocturnal BP oscillations may pave the way toward addressing the future question of whether apnea-induced epochs of BP oscillations mediate lasting cardiovascular anomalies including hypertension [14].

Currently, the measurement of nocturnal BP oscillation is neither part of the clinical diagnostic study of sleep apnea nor part of prognostic studies of treated apnea patients [15], primarily due to the cost and complexity of such measurements. Hence, apnea-triggered oscillations are likely to go unnoticed during clinical diagnostic studies. However, some researchers have previously studied the effect of sleep apnea on nocturnal BP [6, 16–19]. Most of these studies focused primarily on average or overall BP across the night. That is, the temporal scale of integrating and distilling the BP measurements covered the entirety of the night or even 24 hours, rather than considering stages of the sleep and the individual apneic events during the night. Examples of these measures are hourly profile of mean arterial pressure [13] and sequential spectral analysis of 24-hour BP recording which included sleep periods [20]. These approaches do not delineate dynamic BP responses to apnea events and how sleep stage may influence those relationships.

Our prior investigations of nocturnal BP dynamics in OSA patients documented that for almost every obstructive event, the OSA patient experiences a significant surge in BP [21–23]. Since OSA patients experience numerous obstructive apnea and hypopnea events during sleep, their cardiovascular system is subjected to significant BP oscillations. Hence, quantifying these oscillations for the whole night is of interest.

In this paper, we present a method of aggregating and quantifying dynamic changes in beat-by-beat BP elicited by sleep apnea events. The method provides multiple quantitative measures of the severity of apnea-mediated oscillations in nocturnal BP. These measures may in turn serve as possible surrogate measures of sympathetic nervous system responses to apnea events. The findings from the application of the proposed method to overnight beat-to-beat BP recordings from 10 OSA patients are also presented.

## 2. Materials and Methods

### 2.1. Noninvasive Blood Pressure Monitoring

We recorded nocturnal beat-by-beat arterial BP with the Finapres (BMEYE, Amsterdam, Netherlands) [24], which uses a pressurized finger cuff to sense arterial BP in finger arteries, typically the digitus secundus, digitus medius, or digitus quartus. Details and validity of this method are well established and reported [24]. Briefly, the device uses photoplethysmographic feedback to dynamically control the inflation pressure of a finger cuff. The cuff pressure adjustment during each cardiac cycle compensates for the change in arterial blood volume of the finger. Hence, cuff pressure equals BP in the arteries of the finger.

### 2.2. Blood Pressure Waveform Analyses

#### 2.2.1. Quantification Metrics

**Figure 1:**
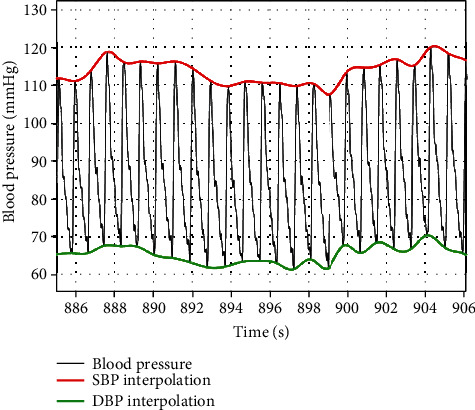
An example of nocturnal systolic and diastolic pressure envelopes derived from beat-to-beat blood pressure waveform measured using Finapres.

Figure 1 illustrates how we extracted peak systolic pressure (SBP) and diastolic pressure nadir (DBP) from each heartbeat using a customized MATLAB (Mathworks, Natick, MA) program. Envelopes of the detected peaks (i.e., SBP) and troughs (i.e., DBP) were generated using cubic spline interpolation (spline function in MATLAB) (Figure 1).

#### 2.2.2. Quantification of Blood Pressure Oscillations

**Figure 2:**
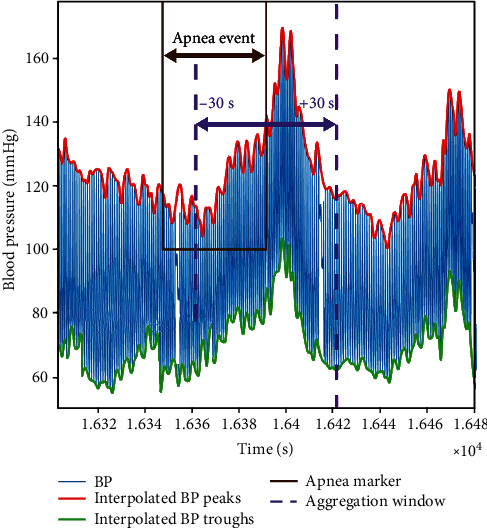
A sample of nocturnal systolic and diastolic pressure envelopes and the mean and pulse pressure curves derived from beat-to-beat blood pressure waveform measured using Finapres.

We observed that BP oscillations tended to be greater in magnitude for obstructive apnea events as compared with hypopneas. Hence, for the present study, we focused our analysis on the effects of apnea events on the BP oscillations; we excluded hypopneas, mixed apneas, and central apneas from the analysis. An important consideration in the quantification of the effect of apnea events on BP is the temporal distance between consecutive events, as when apnea event occurs in rapid succession, the BP oscillations induced by each of the apnea events may be affected by the effects of the preceding and succeeding events. Due to the complexity of isolating the effect of each event when a series of apnea events occur in rapid succession, we concentrated on determining the impact of apnea events that were at least 30 s separated from the preceding and also 30 s from the succeeding apnea event. That is, the apnea events that were selected for this study had at least a 30 s gap between the end of one event and the start of the next event. The rationale for selecting this gap between events is based on examining the data from OSA patients which showed that the peak of averaged SBP values nearly always occurs within a 30 s window post the end of an apnea event. Since the intent of the event selection criteria is to attain an estimate of the level of BP surges due to each apnea individually, this minimum of 30 s gap between events allows one to assess the dynamics of the BP surge in response to a single apnea event. For ease of reference, we refer to events according to this selection criterion as *isolated apneas*. Next, we analyzed the BP metrics for the isolated apneas within a 60 s window that is centered on the end of an apnea event and spans 30 s in both directions from that point.  Figure 2 illustrates this window with arrows that are labeled with -30 s and +30 s spanning between dashed vertical lines.

**Figure 3:**
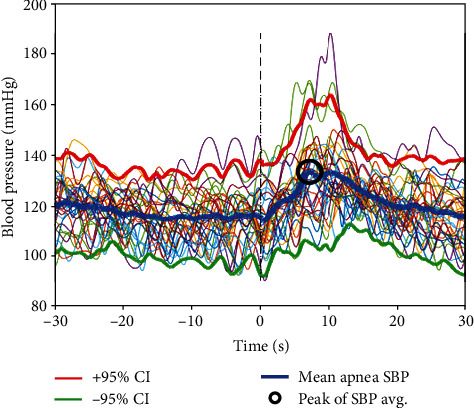
Aggregation of SBP apnea-elicited BP oscillations for a sample subject (34 SBP surges aggregated). The vertical dash line signifies the center of the aggregation window which is the point of alignment SBP of all the isolated apneas for the subject. The abbreviation CI refers to the confidence interval, and the small red circle designates the peak of the mean of the aggregated SBP values.

To arrive at quantitative measures of the BP oscillations that embody the dynamics of BP variations elicited by all of the isolated apnea events throughout the night for each subject, we aligned the SBP (and separately DBP) envelopes for the events. We then centered on the aligned termination point of a window that spanned 30 s forward and 30 s backward in time (Figure 2) and called it *aggregation window*. Within the aggregation window, we computed the sample-by-sample average of the SBP (and separately the DBP) envelopes of BP during the aggregation window for each subject. An example of the application of this method to SBP envelopes of selected apneas from a subject is shown in Figure 3. In this figure, the blue curve represents the mean values of SBP sample points within the aggregation window, while the red and green lines are the ±95% confidence interval (CI) for the aggregated mean values of the blue curve. The black circle marks the peak of the resulting mean SBP curve. The aggregated SBP curve (i.e., blue line) reflects a sample-by-sample average of the SBP values within the aggregation window.

Once the envelopes of the SBP and DBP surges are aggregated, several features of the resulting aggregated SBP and DBP waveforms can be considered to quantify BP oscillations. Of main interest is the peak value of the average waveform (e.g., the small black circle on the blue waveform in Figure 3), as it represents the peak (and temporal location) of the largest value of the averaged SBP (e.g., the blue curve in Figure 3) over the aggregation window. Particularly, it is an average representation of the expected peak of the SBP surge that on the average occurs after isolated apneas. The temporal location of this peak is labeled as *postapnea delay* for ease of reference. The postapnea delay provides an estimate of the expected time point when the apnea-elicited SBP surge peaks. Similar quantification was also applied to the DBP surge envelopes. Additionally, to compare apnea-induced BP oscillations to the BP level when the patient is not experiencing apnea, we identified 60 s segments of sleep with normal breathing when no respiratory event had occurred for each subject. During this interval, the values of SBP and DBP were, respectively, averaged to obtain the *baseline* SBP and DBP for each subject.

**Figure 4:**
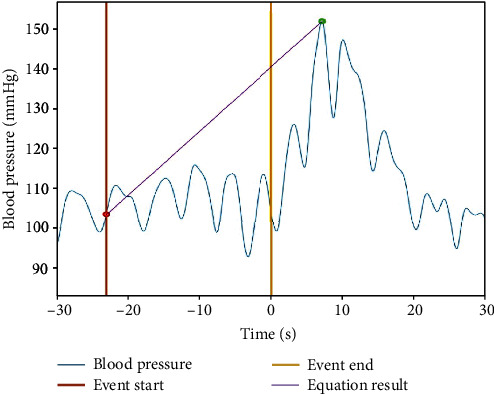
Illustration of the estimation of the rate of rise in blood pressure during an apnea event. The line resulting from fitting equation to the points designated with red and green circles. The blood pressure amplitude at the start of the event (i.e., at red point) has a magnitude that is equal to the average of the SBP value at the start of the event and four preceding sample values of SBP. The small green circle located on the peak of SBP has the magnitude that is equal to the average of the peak SBP together with two preceding and two succeeding SBP samples.

Another measure of interest is a quantitative measure of the rate of rise in the SBP and DBP. For this purpose, we used a linear estimate of the rate of rise by fitting a straight line to two points: (1) the average of five sample values of the SBP envelope at the start of each apnea event and (2) the average of five values centered around the highest SBP surge point elicited by the apnea event (Figure 4). The slope of the fitted line was used as an estimate of the rate of rise. Applying the same method, the rate of rise in DBP due to apnea events was computed using the DBP envelope.

To investigate whether BP metrics proposed in this study are affected by sleep stages, we used the approach described above and computed the baseline SBP and DBP during each sleep stage. For this purpose, we considered both rapid eye movement (REM) and nonrapid eye movement N1, N2, and N3 sleep. Specifically, we grouped isolated apneas for each subject according to the sleep stages in which they occurred. However, to avoid any confounding that may result from inclusion of events that span sleep stage transitions, we chose only those OSA events that occurred in their entirety within a single stage. For determination of possible effect of sleep stages on BP oscillations, the average baseline values of SBP and DBP envelopes were computed for each stage. The method of determining the baseline window for each sleep stage was to identify a 60 s period in each sleep stage which was void of any respiratory event. Then, the corresponding values of SBP and DBP were averaged over this period to obtain the baseline values for that stage. The sleep-stage-specific SBP and DBP baseline values were used to quantify the relative magnitudes of SBP and DBP apnea-elicited surges during those sleep stages.

## 3. Experimental Evaluation

### 3.1. Subjects

We studied ten volunteer OSA patients (8 M and 2 F; BMI: 34.5 ± 7.8 kg/m^2^; age: 53.2 ± 5.7 yr.; and AHI: 63.5 ± 28.7 events/h with a range of 18.3-105.4). Based on AHI, subjects were clinically classified as having moderate to severe OSA. The experimental protocol for this research was approved by the University of Texas at Arlington Institutional Review Board. All subjects signed written informed consent prior to their participation. No adverse event or consequence occurred as a result of our testing.

### 3.2. Experimental Setup

Full-night polysomnography (PSG) was obtained for each subject at a sleep laboratory accredited by the American Academy of Sleep Medicine (Sleep Consultants Inc., Fort Worth, Texas). PSG measures included electroencephalogram (EEG), electrooculogram (EOG), electromyogram (EMG), oral and nasal airflow, chest and abdominal movement, leg movement, snoring, blood oxygen saturation, and video monitoring using the Sandman sleep study system (Natus Medical Inc., Oakville, Ontario, Canada). BP was continuously sensed during the sleep study using Finapres, as described above. Finapres analog output was fed into a data acquisition card (DAQ) (DAQ6024E by National Instruments, Austin, TX, USA) installed in a laptop computer (Dell Latitude E640, Round Rock, TX, USA). The DAQ had a 12-bit analog-to-digital conversion resolution and sampled the BP signal at 1000 Hz. A synchronization signal fed into both the Sandman system and the DAQ board allowed precise alignment of BP and PSG data [18].

### 3.3. Analysis of Experimental Data

A registered polysomnographic technologist scored PSG data according to established standards. They were blind to the objectives of this study. The scored PSG data were synchronized with BP data using the synchronization signal described above. These steps allowed the identification of normal breathing, apneic events, sleep stages, and associated BP variations.

## 4. Results

Subjects spent 7.28 ± 0.66 h (mean ± SD) in bed and slept for 4.77 ± 1.64 h of that time. The mean durations of N1, N2, N3, and REM for the subjects were, respectively, 174.22 ± 77.94, 83.22 ± 61.22, 6.12 ± 8.3, and 29.58 ± 23.88 min.

**Figure 5:**
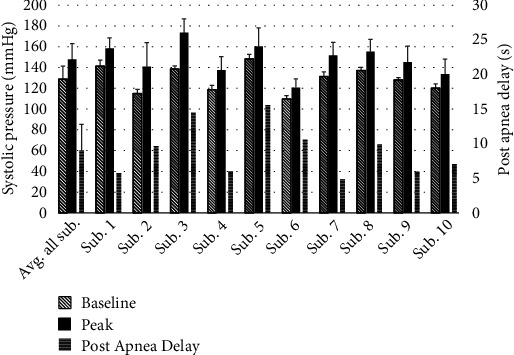
Baseline, surge peak, and postapnea delay mean and standard deviation (error bars) of SBP elicited by apnea events.

Figure 5 shows the average and standard deviation of the baseline, apnea-elicited peak, and postapnea delay of SBP for each subject, obtained by aggregating the systolic blood pressure waveform throughout the night as described earlier. The mean and standard deviation of the said metrics for all the subjects are also shown.

**Figure 6:**
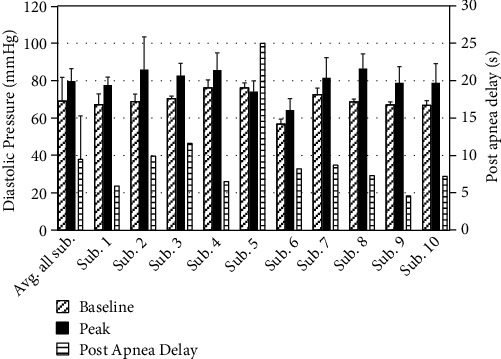
Diastolic blood pressure baseline, peak, and postevent delay means and standard deviation (error bars) for individual and the corresponding average of the means for all subjects.

The means and standard deviations of DBP baseline, apnea-elicited peak, and postevent delay for each subject as well as the average of the means and the associated standard deviation for all subjects as well as the population sample of the subjects are shown in Figure 6.

As explained earlier, to delineate any possible effect of sleep stage on nocturnal BP oscillations elicited by apnea, we also grouped OSA events for subjects according to the sleep stage. However, to avoid any confounding that may result from the inclusion of events that span sleep stage transitions, we chose only those OSA events that occurred in their entirety within a single stage.

As a result of this selection criteria, the selected isolated apneas were not associated with each and every one of the subjects in every sleep stage. For comparing the effect of the selected isolated apneas associated with each stage, we computed the baseline BP values for each subject in the corresponding sleep stage in accordance with the method described earlier.  Table 1 contains the results of the analysis of SBP at various sleep stages. The number of isolated apneas that satisfied the selection criteria of occurring in their entirety within one sleep stage ranged from none to 27 in N1, 13 in N2, and 10 in REM. Compared to the baseline values, the mean peak of SBP surges in N1 ranged from 7.1% to 18.7%, in N2 ranged from 10.4% to 18.7%, and in REM from 3.7% to 8.2%.

Similarly, we computed the variations in DBP due to apnea events in various sleep stages for the subjects. The results are reported in Table 2. Overall, the percentage of mean DBP compared to baseline was higher with 10.2% to 24.8% in N1, 14.1% to 25.2% in N2, and 5.8% to 9.0% in REM.

**Figure 7:**
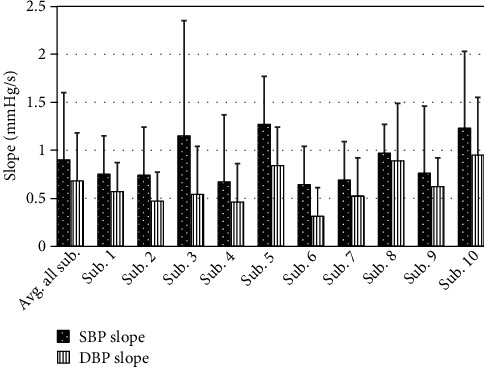
Mean and standard deviation (error bars) of slopes of SBP and DBP surges during apnea events.

The rate of rise in SBP and DBP elicited by isolated apneas for each subject was also computed using the linear slope approximation described earlier (Figure 4). The results are shown in Figure 7. For SBP surges, the average slope for individual subjects ranged from 0.6 to 1.3 mmHg/s, and the mean ± standard deviation for all the subjects combined equaled to 0.9 ± 0.7 mmHg/s. The range of slopes for DBP surges for individual subjects was 0.3 to 1.0 mmHg/s, and the mean and standard deviation for all subjects equaled to 0.6 ± 0.5 mmHg/s. Statistical comparison of the mean rate of rise for SBP and DBP showed that they are significantly different (*p* < 0.02).

## 5. Discussion

The results confirm that OSA events are associated with surges in beat-by-beat BP and further demonstrate the high variability associated with these BP surges. This variability poses a challenge. While quantifying features of every surge trajectory individually is possible, aggregating BP responses as shown in Figure 3 quantifies the overall shape of the combined surges, peak value, and postapnea delay in reaching peak BP for a given study population. The resulting waveform (e.g., blue line in Figure 3) and mathematical underpinning provides magnitude, rate of rise, peak, and postapnea delay with high temporal resolution. The method offers a way of quantifying the combined trend of the response of BP of patients to the stimulation that each apnea induces to the autonomic nervous and cardiovascular systems. Conversely, the derived single aggregated trajectory conceivably facilitates dynamic modeling of apnea-elicited blood pressure surges for individual patients given the appropriate inputs.

By creating an envelope of continuous SBP and DBP data and aligning them at the termination point of apnea events (Figure 2), one can extract the pattern of the BP surge that takes place during and after apneas (Figure 3). This pattern consistently shows that BP surges peak after apnea termination. It is observed that when all apnea-elicited SBP waveforms are aggregated for each subject individually (e.g., Figure 3), the resultant waveforms indicate that all patients experience a net rise in SBP. When the SBP peaks for all subjects are considered (147.68 ± 15.3 mmHg), the coefficient of variation is 10.35%, indicating that the surge values are not widely spread. Further, the coefficient of variation for the subjects' baseline SBP (128.68 ± 12.7 mmHg) was quite comparable at 9.84%.

The rise in DBP for the subjects (Figure 6) was similar to the surge in SBP with the exception of subject 5, where the aggregated DBP waveform resulted in a slightly lower peak value (i.e., 74.1 ± 5.8 mmHg) compared to the baseline DBP (i.e., 76.2 ± 1.9 mmHg). Considering that subject 5 also presented the highest SBP baseline (148.2 mmHg) and lowest percentage rise in SBP (8.2% with an aggregated peak of 160.4 mmHg), the analysis of aggregated DBP waveforms for this subject appears to suggest that when a patient has a relatively elevated baseline BP, the surges in DBP are not as pronounced and may remain approximately the same as the baseline DBP level, while the SBP surges are also on relatively the lower side.

As another outcome of creating an envelope of continuous SBP and DBP data and aligning them at the termination point of apnea events (Figures 2 and 3), one can observe the pattern of the BP surge that takes place during and after apneas (Figure 3). When all apneas for each of the subjects—regardless of the sleep stage in which they occur—are considered, the peak of the aggregated SBP for the patients falls between 4.8 s and 15.6 s with postapnea, and the mean and standard deviation of these postapnea delays in SBP peaks are 9.0 ± 3.8 s (Figure 5). Similarly, the peak of resultant DBP envelopes falls between 4.6 s and 11.6 s; the mean and standard deviation of these postapnea delays in SBP peaks are 9.5 ± 5.8 s (Figure 6).

The postapnea delays correspond to the physiological delay between maximum apnea-induced sympathoexcitation and maximum vascular smooth muscle activation [11, 12]. Further, determining postapnea delays of the aggregated results indicates that much of the pathological cardiovascular strain imposed by sleep apnea occurs outside the temporal boundaries of the respiratory events. The knowledge of the postapnea delays can also be helpful in determining the appropriate duration of a search interval for finding the highest SBP and DBP surge values resulting from apnea events. Referring to the results shown in Figures 5 and 6, the mean and standard deviation of the postapnea delays for the aggregated SDB (9 ± 3.77 s) and DBP (9.49 ± 5.80 s) suggest that using a 30 s window that starts at the alignment point of the BP envelopes (i.e., termination of each event) may be adequate, as the sample mean plus 3 standard deviations of the postapnea delays for SDB (20.3 s) and DBP (26.9 s) remain within a 30 s window post the termination of the apnea (Figure 2).

Considering that autonomic regulation during sleep is affected by the sleep stage [25], it is of interest to explore the possible effect of the sleep stage on apnea-elicited SBP and DBP surges. Since in this paper we have focused on assessing the effects of isolated apneas that occur in their entirety in a single sleep stage and are also separated from apnea by at least 30 s, the number of surges that satisfied the selection criteria within each subject was somewhat limited (Tables 1 and 2). The duration of N1 dominated the sleep stage durations for the subjects (61%), and more subjects had events during N1 that satisfied the selection criteria than other sleep stages. Specifically, 6 out of 10 subjects had events in N1 that satisfied the selection criteria, while only two of the subjects had qualified events in N2 and REM; no event in N3 was qualified based on the selection criteria for any of the subjects.

Referring to subjects that had qualified isolated apneas in multiple stages, subjects 1 and 10 presented qualified events in both N1 and N2. Probing the difference in the surges in N1 and N2 for these subjects, it is observed that subject 1 showed that in N2 compared to N1, there was 2.9% and 3.9% higher surge in SBP and DBP, respectively. Similarly, subject 10 showed 3.9% higher SBP surge in N2 than N1 (Table 1). However, this subject presented only a slight increase (0.4%) in the surge of DBP for N2 compared to N1. Only subject 7 had qualified apnea events in both N1 and REM, and the difference in the percent rise for this subject in REM compared to N1 was 3.2%, with N1 showing a greater surge mean (Table 1). Similarly, DBP surges in subject 7 showed an additional 4.7% rise in N1 compared to REM (Table 2). When postapnea delays for subjects 1 and 10 who presented qualified events in N1 and N2 are considered, it can be observed that the postapnea delay in SBP and DBP for subject 1 was 2.9 s longer in N1, and for subject 10, it was 2.0 s longer. When both postapnea delays and percentages of rise for N1 and N2 in these two subjects are considered, it shows that SBP and DBP crest faster and higher in N2 compared to N1 in both subjects. Of course, to establish whether this is indeed the case in majority of OSA patient population, a larger sample of population needs to be studied in order to have more cases of the same subjects presenting qualified isolated apneas in multiple sleep stages.

Examining the rate of rise of SBP and DBP in the isolated apneas revealed that on the average, SBP is expected to rise at a rate of 0.9 mmHg/s while DBP is expected to rise at an average rate of 0.62 mmHg/s. However, the variations in slopes of SBP and DBP were significant as the coefficients of variation for SBP and DBP rates were 78% and 81%, respectively. Hence, a high degree of variability is present in the rate of rise data.

While the present study provides insight on how individual apnea events affect the overall BP response in the subjects, there are a number of limitations associated with the study. The study focused on isolated apneas. That is, it only considered obstructive sleep apnea events that were separated from other respiratory events by at least 30 s. Further, other types of respiratory events were not included in the study. It is intended that our future studies would eliminate these constraints on the selection of the events. In this regard, hypopneas probably elicit surges in SBP and DBP similar to apneas, albeit of lesser magnitude. If this proves to be true, the number of greater BP peaks across the night for a given OSA patient must approach or approximate their AHI. In our future studies, we plan to examine the effect of hypopnea as well as central and mixed apnea events on BP oscillations and explore possible correlation of the number of the BP surges with AHI. A further refinement would be to examine whether the patient's position when a respiratory event takes place has any effect on the metrics introduced in this paper. Given that the participating subjects had moderate to severe sleep apnea with the mean AHI value for the subjects being 63.5 events/h, the sleep architecture for the subjects was dominated by N1 sleep (approximately 61%). To be able to extend the results, future studies should include subjects with different sleep architecture who may have less severe apnea. It is important to note that the sample size was limited, and in order to extend the findings to broader population, future studies need to include a larger sample size. Among such possible extensions is exploring whether there is any association between the trajectory of the apnea-elicited BP surges and the duration of respiratory events.

## 6. Conclusion

Aggregation of continuously recorded nocturnal BP in OSA patients using the presented method enables highly granular characterization of apnea-mediated surges in nocturnal BP. Application of the method to a sample OSA patient population confirmed that BP surges peak seconds after each OSA event ends. However, the magnitude of SBP and DBP surges, postapnea delays, and rate of rise of BP vary between sleep stages. The collective findings facilitate mathematical modeling of nocturnal BP surges mediated by sleep-disordered breathing events.

## Figures and Tables

**Table 1:** Apnea-elicited variations in SBP during various sleep stages.

	Sleep stage (no. of OSA events analyzed)	Mean ± STD baseline SBP (mmHg)	Mean ± STD peak SBP during apnea (mmHg)	Difference of mean peak and baseline BP (mmHg)	% difference of mean peak and baseline SBP	Postapnea delay (s)
1	N1 (7)	121.4 ± 4.1	130.4 ± 8.5	9.1	7.5%	10.0
1	N2 (12)	116.7 ± 5.4	128.8 ± 12.4	12.1	10.4%	7.1
4	N1 (25)	156.9 ± 8.9	170.5 ± 12.6	13.6	8.7%	11.7
5	N1 (27)	122.1 ± 5.9	137.3 ± 13.8	15.2	12.4%	6.3
6	REM (10)	160.2 ± 3.4	166.1 ± 15.5	5.9	3.7%	25.1
7	N1 (5)	109.5 ± 4.8	122.1 ± 6.2	12.6	11.5%	7.4
7	REM (6)	109.9 ± 3.6	118.9 ± 8.4	9	8.2%	7.1
8	N1 (12)	135.7 ± 6.6	152.2 ± 9.7	16.5	12.2%	8.5
10	N1 (7)	124.3 ± 6.7	142.8 ± 17.3	18.4	14.8%	5.4
10	N2 (13)	128.6 ± 8.1	152.6 ± 15.0	24	18.7%	3.4

**Table 2:** Apnea-elicited variations in DBP during various sleep stages.

Subject no.	Sleep stage (no. of OSA events analyzed)	Mean ± STD baseline DBP (mmHg)	Mean ± STD peak DBP during apnea (mmHg)	% difference of mean peak and baseline DBP	Postapnea delay (s)
1	N1 (7)	69.3 ± 2.8	76.4 ± 7.1	10.2%	10.0
1	N2 (12)	68.3 ± 3.6	77.9 ± 7.8	14.1%	7.1
4	N1 (25)	75.0 ± 4.1	82.8 ± 7.1	10.4%	11.7
5	N1 (27)	74.9 ± 3.5	84.2 ± 9.4	12.4%	6.3
6	REM (10)	71.1 ± 1.6	75.3 ± 4.3	5.8%	25.1
7	N1 (5)	57.5 ± 3.0	65.4 ± 6.9	13.7%	7.4
7	REM (6)	57.9 ± 2.0	63.1 ± 8.2	9.0%	7.1
8	N1 (12)	71.0 ± 5.2	83.0 ± 9.0	16.9%	8.5
10	N1 (7)	64.1 ± 4.9	80.0 ± 9.3	24.8%	5.4
10	N2 (13)	65.9 ± 5.7	82.4 ± 8.7	25.2%	3.4

## Data Availability

For requests for data and statistical analysis code, please contact the corresponding author.
